# Isolation of a widespread giant virus implicated in cryptophyte bloom collapse

**DOI:** 10.1093/ismejo/wrae029

**Published:** 2024-02-24

**Authors:** Helena H Vieira, Paul-Adrian Bulzu, Vojtěch Kasalický, Markus Haber, Petr Znachor, Kasia Piwosz, Rohit Ghai

**Affiliations:** Department of Aquatic Microbial Ecology, Institute of Hydrobiology, Biology Centre of the Czech Academy of Sciences, 37005 České Budějovice, Czech Republic; Department of Aquatic Microbial Ecology, Institute of Hydrobiology, Biology Centre of the Czech Academy of Sciences, 37005 České Budějovice, Czech Republic; Department of Aquatic Microbial Ecology, Institute of Hydrobiology, Biology Centre of the Czech Academy of Sciences, 37005 České Budějovice, Czech Republic; Department of Aquatic Microbial Ecology, Institute of Hydrobiology, Biology Centre of the Czech Academy of Sciences, 37005 České Budějovice, Czech Republic; Department of Aquatic Microbial Ecology, Institute of Hydrobiology, Biology Centre of the Czech Academy of Sciences, 37005 České Budějovice, Czech Republic; Department of Fisheries Oceanography and Marine Ecology, National Marine Fisheries Research Institute, 81-332 Gdynia, Poland; Department of Aquatic Microbial Ecology, Institute of Hydrobiology, Biology Centre of the Czech Academy of Sciences, 37005 České Budějovice, Czech Republic

**Keywords:** cryptophytes, freshwater giant viruses, giant viruses, Imitervirales, NCLDVs, Nucleocytoviricota, phytoplankton spring bloom, viral isolation

## Abstract

Photosynthetic cryptophytes are ubiquitous protists that are major participants in the freshwater phytoplankton bloom at the onset of spring. Mortality due to change in environmental conditions and grazing have been recognized as key factors contributing to bloom collapse. In contrast, the role of viral outbreaks as factors terminating phytoplankton blooms remains unknown from freshwaters. Here, we isolated and characterized a cryptophyte virus contributing to the annual collapse of a natural cryptophyte spring bloom population. This viral isolate is also representative for a clade of abundant giant viruses (phylum *Nucleocytoviricota*) found in freshwaters all over the world.

## Introduction

The phytoplankton spring bloom is an annually recurring global ecological phenomenon across freshwater and marine habitats [1]. In freshwaters, at the onset of spring, increasingly favourable environmental factors and absence of zooplanktonic grazers create ideal conditions for a surge in photosynthetic activity, mainly by cryptophytes and diatoms. Exhaustion of nutrients (e.g. phosphorus) together with mortality inducing agents such as zooplankton, viruses and parasites have been proposed to cause the collapse of the spring blooms. Although there are multiple reports on the importance of zooplankton grazing [1], the role of parasites (especially viruses) in bloom collapse remains relatively unknown. Recently, during a high-frequency sampling campaign of a freshwater spring bloom of cryptophytes, we recovered hundreds of genomic fragments originating from giant viruses, implicating them in termination of the bloom [2]. Numerous giant virus isolates have been obtained from diverse protist lineages e.g. amoebas [3], haptophytes [4], bicosoecids, [5] and many others [6]. However, despite the widespread distribution of cryptophytes [7], availability of cultures (e.g. the well-known cryptophytes *Rhodomonas*, *Cryptomonas*), and even prior observations of virus infected cryptophytes [8, 9], there is only a single, decade old report of a giant virus isolated from the marine cryptophyte *Teleaulax amphioxeia,* but no genome sequence is available [10]. In order to elucidate the role of giant viruses in the collapse of the cryptophyte bloom, we isolated and characterized for the first time, the genome of a giant virus infecting the common cryptophyte *Rhodomonas*. We subsequently recovered very similar viral genomes from metagenomic datasets from North American and European temperate freshwater habitats.

## Results and Discussion

**Figure 1:**
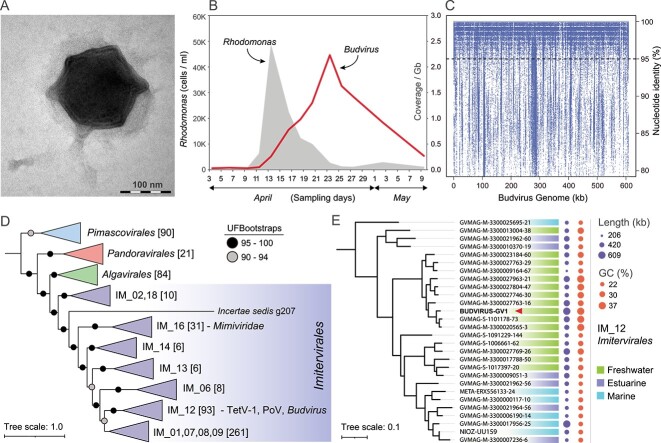
General features of the cryptophyte-infecting *Budvirus*. (A) TEM image of one assembled *Budvirus* particle (ca. 200 nm). (B) Abundance estimates of *Rhodomonas* (cells/ml) and *Budvirus* (genome coverage/gb) during a high-resolution sampling of a spring bloom at Římov reservoir (April–May 2018). Sampling dates are indicated along the x-axis. (C) Nucleotide-level comparison of metagenomic reads from high-resolution sampling of Římov reservoir compared to the *Budvirus* genome. Only reads from the dataset with peak abundance are shown (epilimnion, HRS-ES9; >100 bp alignment length and e-value <1e-5). The Y-axis indicates the percentage nucleotide identity and a dashed line marks 95% identity. (D) Maximum-likelihood phylogenomic tree of NCLDV genomes (n = 662) using seven conserved marker genes. The tree was rooted using orders *Chitovirales* and *Asfuvirales* from the *Pokkesviricetes* class (not shown). Abbreviations: TetV-1, *Tetraselmis* virus; PoV, *Pyramimonas* virus. (E) Subtree from the IM_12 clade of *Imitervirales* showing *Budvirus* and closely related giant viral genomes. This subclade does not include TetV-1 and PoV viruses. The habitat of origin of each genome is colour coded (freshwater, estuarine, marine). Filled circles indicate genome length and GC content. All branches in the subtree are strongly supported (UFB ≥ 96) (details in Supplementary Fig. S5).

We infected a culture of *Rhodomonas lacustris* (isolate NIVA 8/82) with a filtrate of natural lake water collected during a spring bloom (See Supplementary Information). *Rhodomonas* was chosen because it is the predominant blooming cryptophyte at the sampling site (Římov reservoir, Czechia) [2]. We observed loss of culture pigmentation and decreases in cell counts in multiple infected cultures suggesting lysis and the presence of active viruses. Additionally, we inoculated the viral isolate in other cryptophyte cultures (*Rhinomonas*, *Storeatula*, *Teleaulax*, and *Cryptomonas*), which did not result in the collapse of any of the strains. This outcome suggests a host-specific interaction between the infecting virus and *Rhodomonas.* Visualization by electron microscopy revealed the presence of large icosahedral capsids of ca. 200 nm diameter typical for giant viruses (Fig. 1A). Subsequently, larger *Rhodomonas* culture volumes were infected to obtain viral biomass for genome sequencing (See Supplementary Information). Using a hybrid assembly protocol we recovered a complete linear genome of ca. 600 kb with terminal inverted repeats (TIRs) of ca. 4.4 kb at each end, which is common for viruses of the phylum *Nucleocytoviricota* (also known as Nucleocytoplasmic Large DNA Viruses - NCLDVs) [11]. The GC content of these TIRs is ca. 34%, slightly lower than the whole genome (36.7%). Both TIRs also overlap with a gene encoding a chaperone of endosialidase found frequently in bacteriophage tail proteins [12]. Genes encoded in the TIR regions have been reported at least once before [11]. The 5’ TIR has an additional duplication of 46 bp (Supplementary Fig. S1). Hereafter, we will refer to this viral isolate as *Budvirus* after the town of České Budějovice (or Budweis) in the vicinity of the sampling site.

The *Budvirus* genome encodes 474 protein coding genes and three tRNAs (Asn, Ile, and Leu) (Supplementary Tables S1 and S2). Similar to other giant viruses [13], four different genes encoding major capsid proteins between 411 and 487 amino acids long were identified and modelled together with the minor capsid protein (Supplementary Fig. S2, Supplementary Table S3). A large complement of genes involved in transcription and replication were also found (Supplementary Tables S1 and S2). We were able to assign a function to ca. 50% of predicted genes (244 remained hypothetical). However, only three genes involved in protein translation (two initiation factors and a tRNA adenosine deaminase) could be identified. Additionally, it appears that similar to other NCLDVs, *Budvirus* encodes several components of the ubiquitin-proteasome system (UPS) (Supplementary Table S2), e.g. both E3 ligase and ubiquitin were found that may play a role in degrading host protein machinery.

Relative abundance of the *Budvirus* genome during a cryptophyte spring bloom was assessed by comparison against a high-resolution sampling metagenomic dataset (from the same site of isolation of *Budvirus*) (Fig. 1B). The abundance of *Budvirus* was found to continuously increase with the concomitant decline in the population of *Rhodomonas*. This shows that *Budvirus* is abundant at the sampling site and likely contributes towards the collapse of the blooming *Rhodomonas* population. Moreover, examination of data from annual timelines of monthly *Rhodomonas* cell counts (Supplementary Table S4) and metagenomes also suggested the periodic appearance of this viral genome at the sampling site (Supplementary Fig. S3). The abundance of the *Budvirus* genome is also evident in the fragment recruitment plot at maximal abundance during the high-resolution sampling timeline (Fig. 1C). The distribution of metagenomic reads across the entire genome suggests presence of multiple, closely related viral lineages infecting *Rhodomonas.* Moreover, there appears to be little evidence of any substantial metaviromic islands in the genome (i.e. genomic regions with extremely low coverage in metagenomes in comparison to most of the genome). We found only three genes that appeared to be of very low coverage when the genome was compared to freshwater metagenomes (Supplementary Fig. S4). However, no function could be predicted for any of these genes. This implies that differences between closely related viral genomes during the spring bloom are more due to differences within the genes themselves, e.g. single nucleotide polymorphisms (SNPs) rather than gene content (Supplementary Table S5). Even though function prediction was possible for some genes with the most SNPs (Supplementary Table S5), no obvious inferences could be drawn.

Phylogenomic analyses based on a conserved set of seven marker genes across a comprehensive collection of NCLDV genomes [13] placed *Budvirus* into the order *Imitervirales*, family IM_12 (Fig. 1D and E, Supplementary Fig. S5, Supplementary Table S6). Most members in this clade are recovered from metagenomes (Giant Virus Metagenome Assembled Genomes or GVMAGs). This clade also contains two viral isolates, *Tetraselmis* virus (TetV-1) and *Pyramimonas* virus (PoV) both infecting chlorophytes (Supplementary Table S6) [14, 15]. Even though they are placed together in clade IM_12, there was no significant nucleotide identity between *Budvirus* and these two viral isolates and average amino acid identities were very low (38%–39%). However, several other GVMAGs appear to be closely related to *Budvirus* (Fig. 1E). All-vs-all comparisons at nucleotide and protein level between genomes related to *Budvirus* recapitulated groups similar to those observed in the phylogenomic analyses (Supplementary Figs S6 and S7). All neighbours of *Budvirus* identified by phylogenomic analyses are derived from metagenomic assemblies, and none are recovered in a single contig, making *Budvirus* an ideal representative with the only complete genome in this entire clade (Supplementary Table S6).

**Figure 2:**
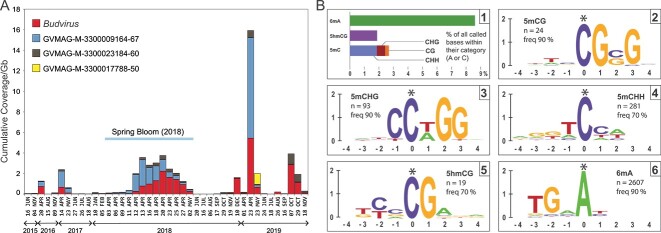
(A) Abundance of giant viral genomes in the *Budvirus* clade in the Řimov reservoir metagenomic timeline (epilimnion datasets only). Cumulative abundances (in coverage/Gb) are shown on the y-axis and date of sampling is indicated in the x-axis. Legend for each viral genome is shown at the top left. The time period of high-resolution sampling in 2018 is marked by a line (1st week of April 2018-1st week of May, 2018). (B.1) Proportion of bases with modifications across all called bases within the same category (A or C) (details in Supplementary Table S7). (B.2–6) Sequence logos generated for positions with a high frequency of methylated/hydroxymethylated base calls: 6 mA = 6-methyladenine, 5hmCG = 5-hydroxymethylcytosine (CG-context), 5mC = 5-methylcytosine, 5mCG - 5-methylcytosine (CG-context), 5mCHG - 5-methylcytosine (CHG context; H = A,T or C), 5mCHH - 5-methylcytosine (CHH context).

Nearly all phylogenomic neighbours of *Budvirus* are derived from metagenomic datasets from freshwater lakes or estuarine waters suggesting their far higher prevalence in freshwaters (Fig. 1E). To test this hypothesis, we assessed the abundance of the entire clade across freshwater metagenomic datasets from all over the world. These analyses showed that *Budvirus* is the most abundant giant viral genome across the freshwater metagenomic datasets examined here, closely followed by two GVMAGs (originating from Lake Simoncouche, Canada and Lake Michigan, USA) (Supplementary Fig. S8). Significantly, all these viral genomes are also abundant both in lakes in Europe and North America which indicates that this newly isolated virus is a representative of a globally abundant and ecologically important group of viruses. When we examined the abundance of related viral genomes in the Římov reservoir itself (the isolation site for *Budvirus*) we found that the closely related viral genome (GVMAG-M-3300009164-67, assembled from lake Simoncouche) also showed similar abundances to *Budvirus* and peaked even earlier than *Budvirus* during the spring bloom suggesting it could also infect *Rhodomonas* (Fig. 2A, Supplementary Fig. S9). These observations further support the global distribution of the *Budvirus* clade and related neighbours in freshwaters.

Genomic DNA methylation is widespread across prokaryotes, eukaryotes and viruses, as it influences both gene regulation and confers protection against foreign DNA [16]. It has been recently shown that NCLDV genome methylation is not universal [17], (e.g. *Mimiviridae* and *Pithoviruses*). However, we found multiple lines of evidence for different types of methylations in the *Budvirus* genome. A total of 12 DNA methylases were identified, of which seven are predicted to methylate adenine (6 mA), four cytosine (5mC), and one without a known target (Supplementary Table S7). DNA sequence recognition sites were already known for at least eight of these methylases. Only two restriction endonucleases were encoded in the genome. Both these endonucleases were predicted to cut at the sites of cytosine methylation (5’-CGCG-3′ and 5’-GGNCC-3′) protected by two encoded cytosine methylases (Supplementary Table S7).

We also detected methylated bases by basecalling raw long-reads against the assembled *Budvirus* genome (Fig. 2B, Supplementary Fig. S10). Adenine methylation appears widespread at the population level, with 2607 sites being methylated in ≥90% of called reads, compared to only 145 C sites. We were also able to detect different sequence contexts of cytosine methylations (Supplementary Fig. S10). Moreover, by examining the flanking sequences of the predicted methylated sites, we reconstructed potential DNA methylation sequence patterns for all these base modifications (Fig. 2B). In concordance with the sites predicted for two cytosine methylases and restriction endonucleases (Supplementary Table S3), we could reconstruct the same sites from sequence logo analyses (Fig. 2B.2 and 2B.4).

There are a total of five adenine methylases with a predicted site but no restriction enzymes with a cognate site were found. Sequence logo analyses revealed a strong preference for A methylation at 5’-TGHA-3′ that was further refined to TGCA after raising the modified fraction threshold to ≥98% (n = 102; Supplementary Fig. S11). This site (i.e. TGCA) was also predicted to be the modification site of the methylase encoded by the gene cds156 (Supplementary Table S3). Given that adenine methylation appears widespread and there are no restriction endonucleases predicted for these modified sites, it is likely that this is a protective mechanism against host restriction endonucleases that likely target these sites.

Here, we isolated and analysed the genome of a giant virus of the phylum *Nucleocytoviricota* infecting the unicellular freshwater cryptophyte *R. lacustris*. *Budvirus* is a representative of a globally important group of viruses that likely contributes to spring phytoplankton bloom collapse in freshwater lakes. This newly established *Budvirus-Rhodomonas* system represents an ecologically relevant model for further studies of the life strategies of such viruses, the mechanisms of host-virus interactions and their effect on the evolutionary history of these ecologically relevant photosynthetic protists.

## Supplementary Material

Supplementary_Figure_S1

Supplementary_Figure_S2

Supplementary_Figure_S3

Supplementary_Figure_S4

Supplementary_Figure_S5

Supplementary_Figure_S6

Supplementary_Figure_S7

Supplementary_Figure_S8

Supplementary_Figure_S9

Supplementary_Figure_S10

Supplementary_Figure_S11

Supplementary_Table_S1_wrae029

Supplementary_Table_S2_wrae029

Supplementary_Table_S3_wrae029

Supplementary_Table_S4_wrae029

Supplementary_Table_S5_wrae029

Supplementary_Table_S6_wrae029

Supplementary_Table_S7_wrae029

Supplementary_Information

Supplementary_Information_wrae029

## Data Availability

The sequencing data are available in EBI-ENA within Bioproject PRJEB6583. The *Budvirus* genome assembly was deposited in EBI-ENA under the accession GCA_963556535. Nanopore MinION long-read data was deposited under experiment number ERX11398554 (Run ERR12014250). Illumina data was deposited with experiment accession number ERX11398555 (Run ERR12014251). Additional data on capsid structural predictions, methylation calls, phylogenomic tree construction and assembly reconciliation is supplied in the Figshare repository at 10.6084/m9.figshare.24081015.
