# Supplementary material for: Isolation of a widespread giant virus implicated in cryptophyte bloom collapse
Source: ISME J. 2024 Feb 24;18(1):wrae029. doi: 10.1093/ismejo/wrae029 (PMC10960955; doi:10.1093/ismejo/wrae029)
Supplement: Supplementary_Figure_S1 [file supplementary_figure_s1.pdf]

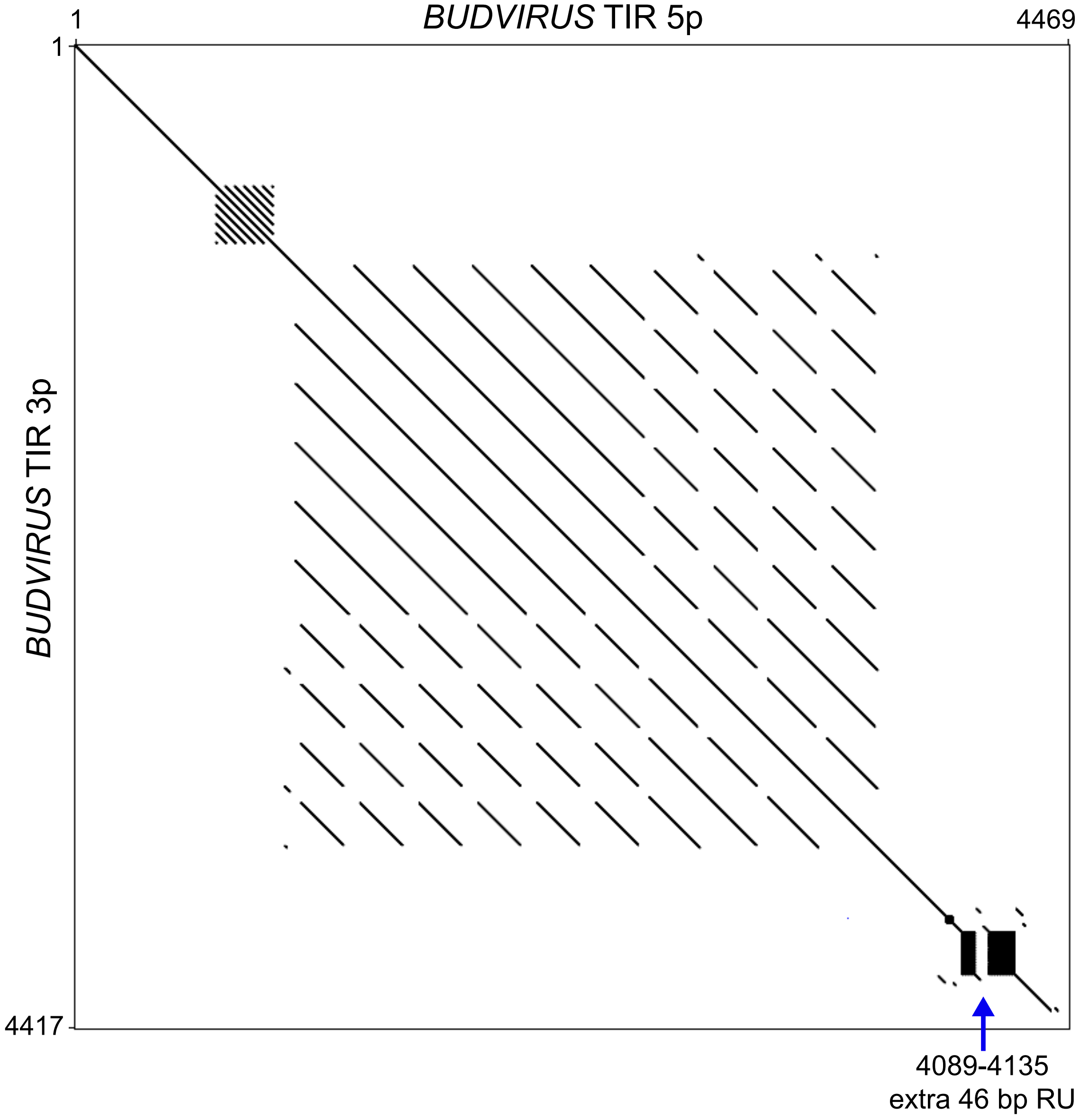

Supplementary Figure S1. DNA dot plot of *Budvirus* terminal inverted repeats (TIRs).
The TIR present at the beginning of the genome (5p TIR) was reverse complemented for comparison. A region corresponding to the extra repeat unit (RU) present in the 5 prime TIR is marked with a blue arrow. Plot made using the Gepard 2.1 software (window size = 50 nt, word size = 50 nt).
