# Supplementary material for: Isolation of a widespread giant virus implicated in cryptophyte bloom collapse
Source: ISME J. 2024 Feb 24;18(1):wrae029. doi: 10.1093/ismejo/wrae029 (PMC10960955; doi:10.1093/ismejo/wrae029)
Supplement: Supplementary_Figure_S2 [file supplementary_figure_s2.pdf]

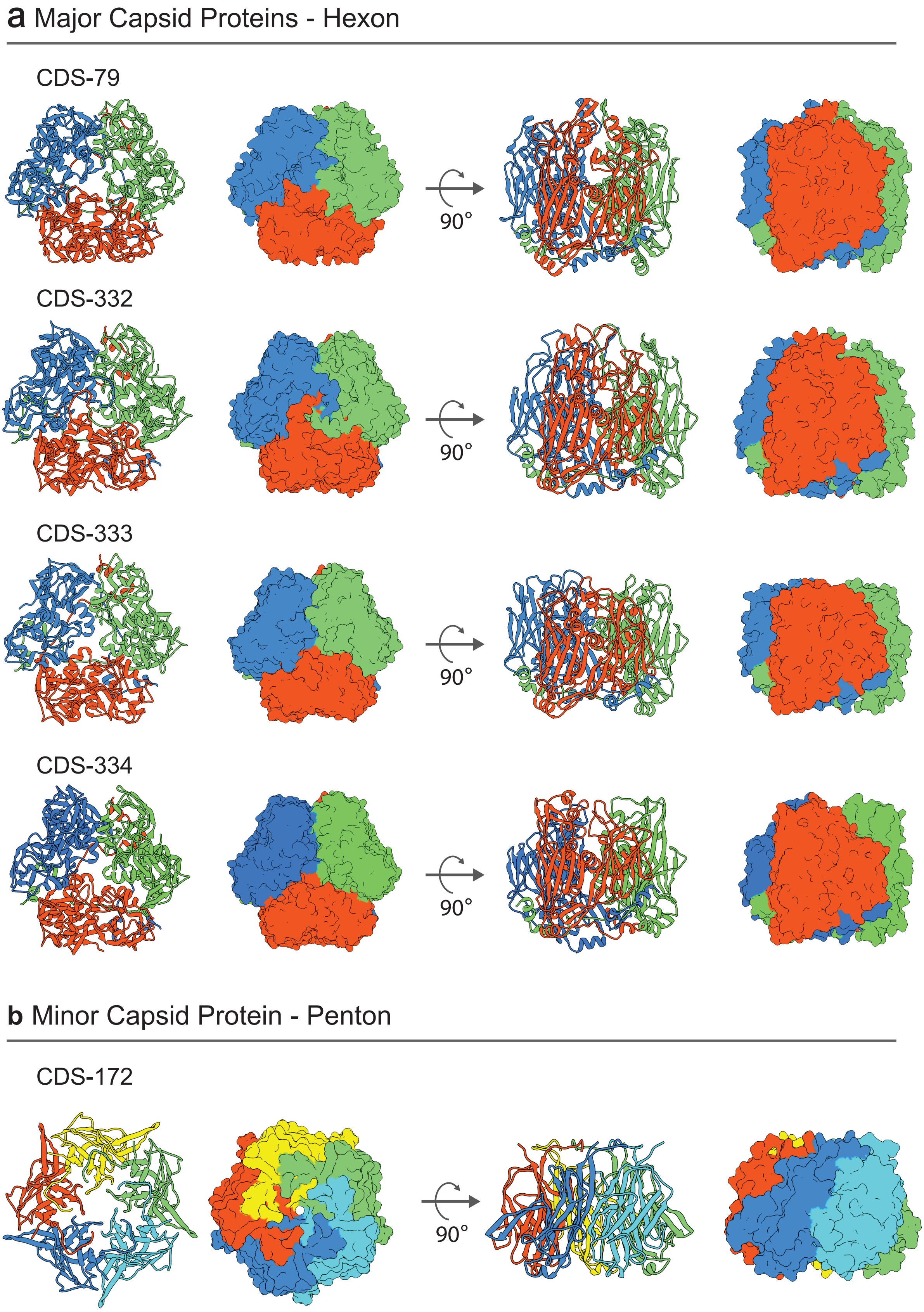

Supplementary Figure S2. *Budvirus* modelled capsid proteins.
a. Hexon trimer as seen from outside the capsid and in a side view. Four different genes encode complete major capsid proteins in this virus. **b.** Penton pentamer as seen from outside the capsid and in a side view. Models for both types of capsids are shown as ribbons and with surface view.
