# Supplementary material for: Isolation of a widespread giant virus implicated in cryptophyte bloom collapse
Source: ISME J. 2024 Feb 24;18(1):wrae029. doi: 10.1093/ismejo/wrae029 (PMC10960955; doi:10.1093/ismejo/wrae029)
Supplement: Supplementary_Figure_S3 [file supplementary_figure_s3.pdf]

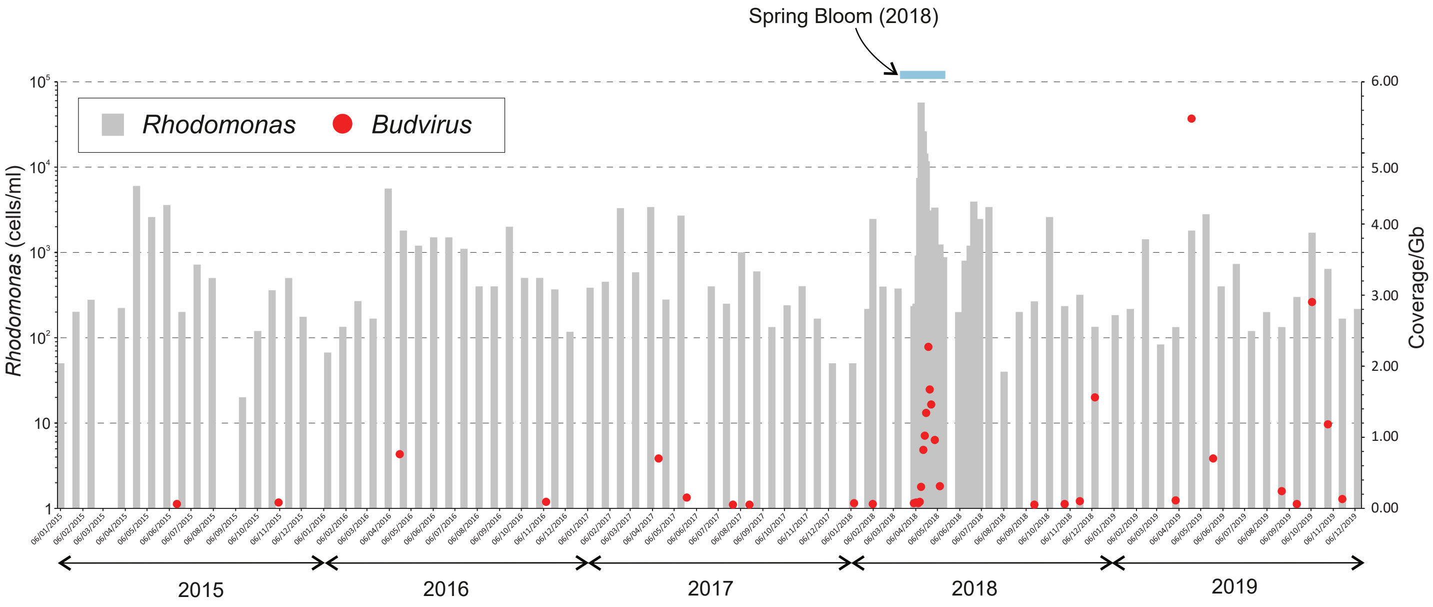

Supplementary Figure S3. Long term abundance of *Rhodomonas* by microscopy (left y-axis, cells/ml, logarithmic scale) and *Budvirus* (right y-axis, coverage/gb) from 2015-2019 in the Řimov reservoir. The high-resolution bloom campaign is indicated with a blue line (1st week of April 2018-1st week of May, 2018).
