# Supplementary material for: Isolation of a widespread giant virus implicated in cryptophyte bloom collapse
Source: ISME J. 2024 Feb 24;18(1):wrae029. doi: 10.1093/ismejo/wrae029 (PMC10960955; doi:10.1093/ismejo/wrae029)
Supplement: Supplementary_Figure_S4 [file supplementary_figure_s4.pdf]

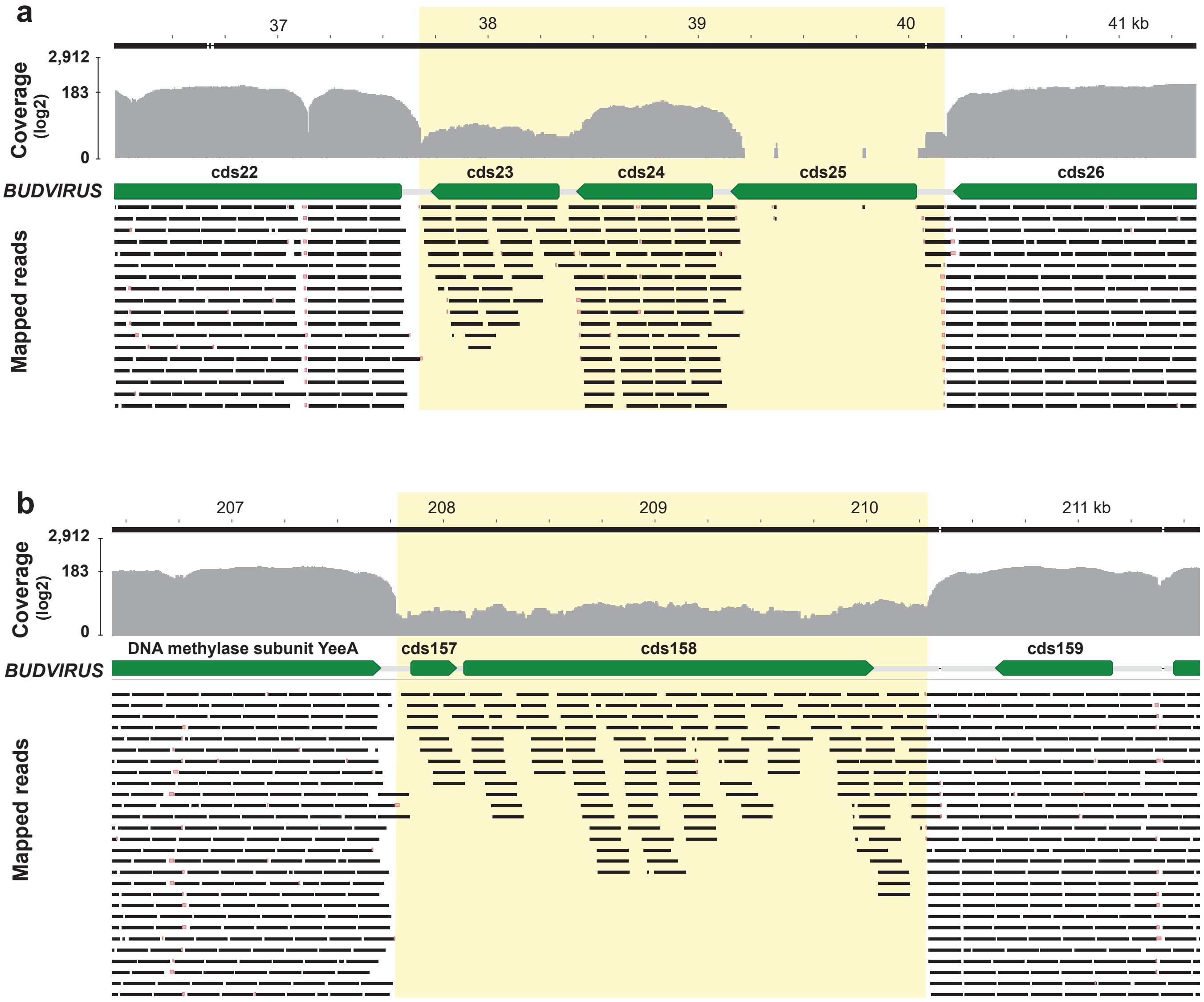

Supplementary Figure S4. Genomic islands identified in environmental populations of *Budvirus* from the Římov freshwater reservoir (Czech Republic). **a.** First genomic island (~ 2.5 kb) consisting of three genes with different coverage levels with respect to the genome median coverage as well as between themselves. **b.** Second genomic island (~ 2.5 kb) containing two genes with lower coverage than the median value of the *Budvirus* genome. Genes are colored green and genomic islands are highlighted in yellow. The coverage scale is logaritmic with the median value (183) indicated between minimum and maximum.
