# Supplementary material for: Isolation of a widespread giant virus implicated in cryptophyte bloom collapse
Source: ISME J. 2024 Feb 24;18(1):wrae029. doi: 10.1093/ismejo/wrae029 (PMC10960955; doi:10.1093/ismejo/wrae029)
Supplement: Supplementary_Figure_S5 [file supplementary_figure_s5.pdf]

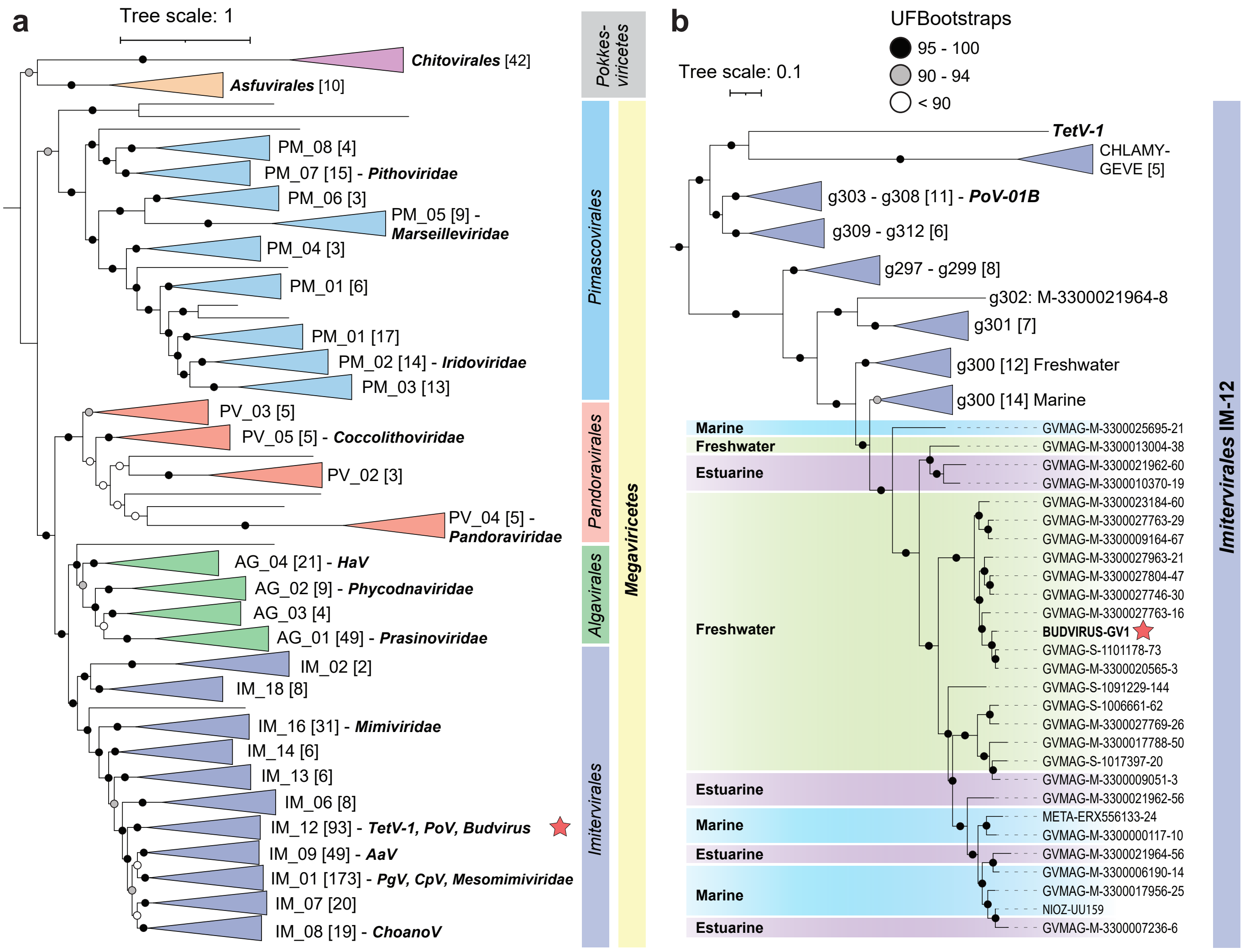

Supplementary Figure S5. Giant virus phylogenomics.

a. Comprehensive maximum-likelihood phylogenomic tree of *Nucleocytoviricota* (NCLDV) including the isolated *Budvirus*. *Chitovirales* and *Asfuvirales* orders from the *Pokkesviricetes* class were used as outgroups. *Budvirus* was placed within order *Imitervirales*, family IM_12, together with two viruses isolated from *Chlorophytes*: *Tetraselmis* virus (TetV-1) and *Pyramimonas* virus (PoV). Other abbreviations: Aav, *Aureococcus anophagefferens* virus; Choan-oV1, *Choanoflagellate* virus; CpV, *Chrysochromulina parva* virus; HaV, *Heterosigma akashiwo* virus; PgV, *Phaeo-cystis globosa* virus. **b.** Expanded IM_12 clade of Imitervirales showing *Budvirus* and related giant virus genomes. The habitat of origin of each genome is colour coded (Freshwater, Estuarine, Marine). Ultrafast bootstraps (UFB) values are encoded by circles, according to the legend in panel **b**.
