# Supplementary material for: Isolation of a widespread giant virus implicated in cryptophyte bloom collapse
Source: ISME J. 2024 Feb 24;18(1):wrae029. doi: 10.1093/ismejo/wrae029 (PMC10960955; doi:10.1093/ismejo/wrae029)
Supplement: Supplementary_Figure_S6 [file supplementary_figure_s6.pdf]

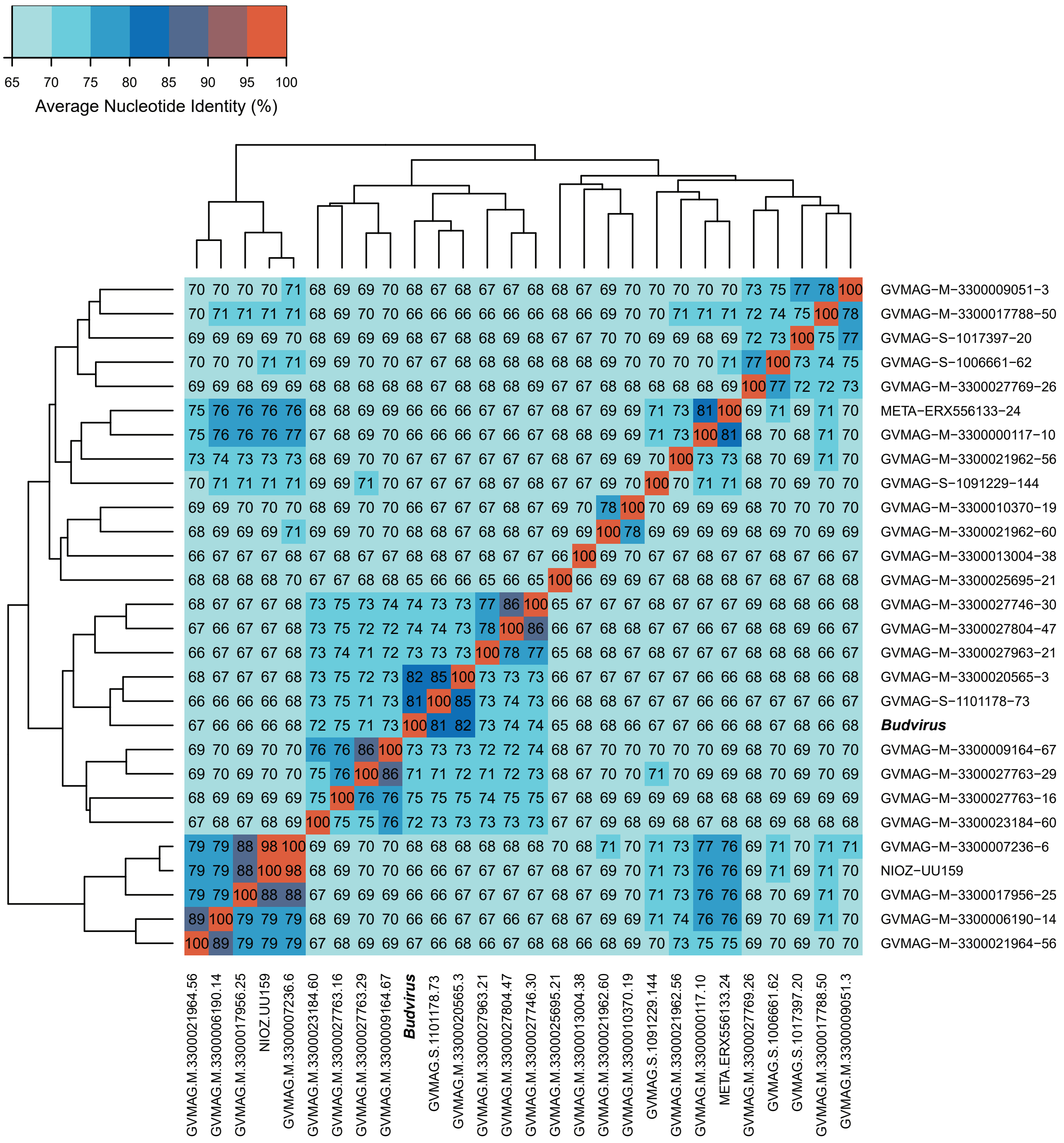

Supplementary Figure S6. All-vs-all average nucleotide percentage identity between *Budvirus* and its phylogenetic neighbors. An identity (%) color scale is shown at top left.
