# Supplementary material for: Isolation of a widespread giant virus implicated in cryptophyte bloom collapse
Source: ISME J. 2024 Feb 24;18(1):wrae029. doi: 10.1093/ismejo/wrae029 (PMC10960955; doi:10.1093/ismejo/wrae029)
Supplement: Supplementary_Figure_S7 [file supplementary_figure_s7.pdf]

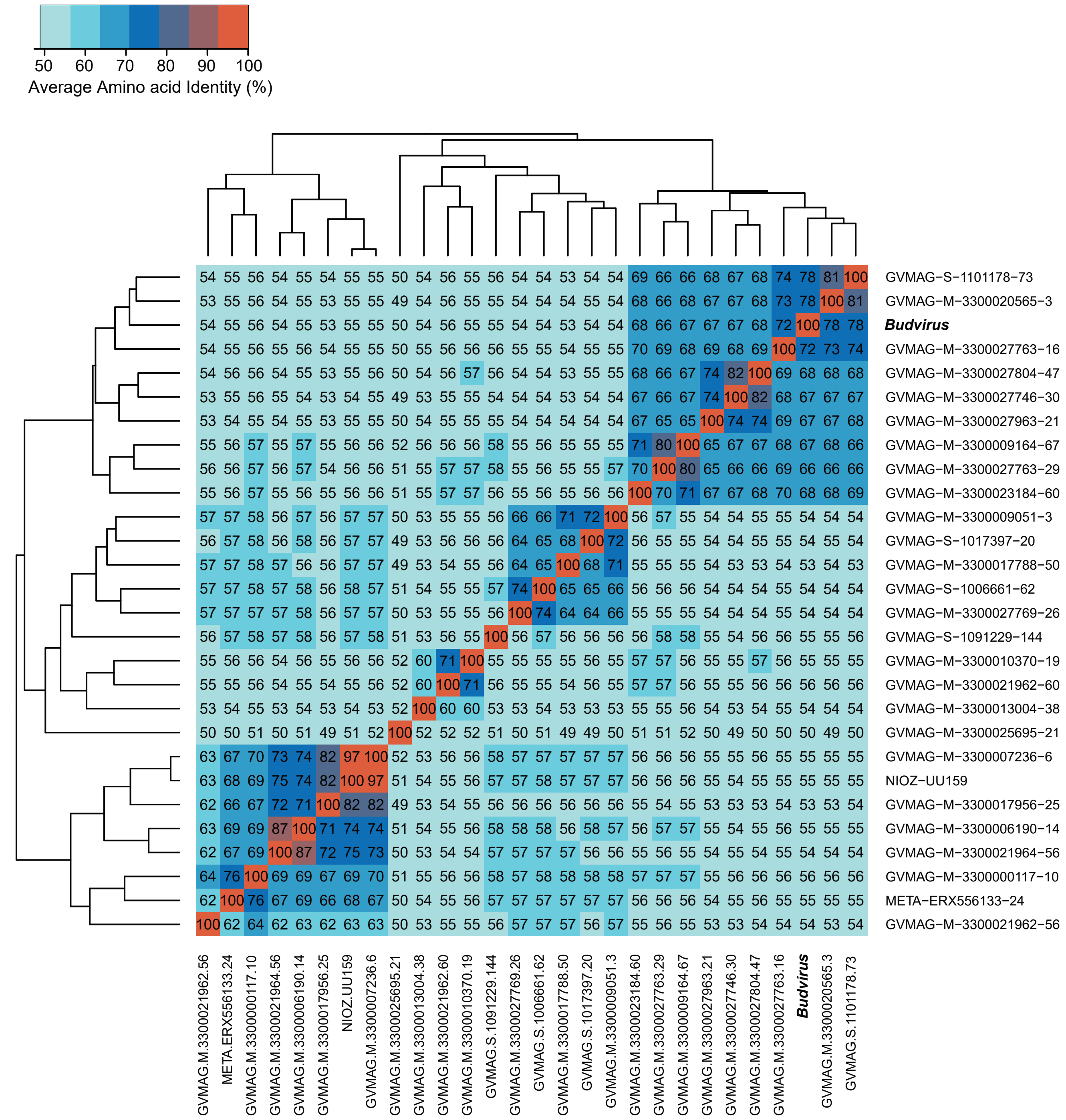

Supplementary Figure S7. All-vs-all average amino acid percentage identity between *Budvirus* and its phylogenetic neighbours. An identity (%) color scale is shown at top left.
