# Supplementary material for: Isolation of a widespread giant virus implicated in cryptophyte bloom collapse
Source: ISME J. 2024 Feb 24;18(1):wrae029. doi: 10.1093/ismejo/wrae029 (PMC10960955; doi:10.1093/ismejo/wrae029)
Supplement: Supplementary_Figure_S8 [file supplementary_figure_s8.pdf]

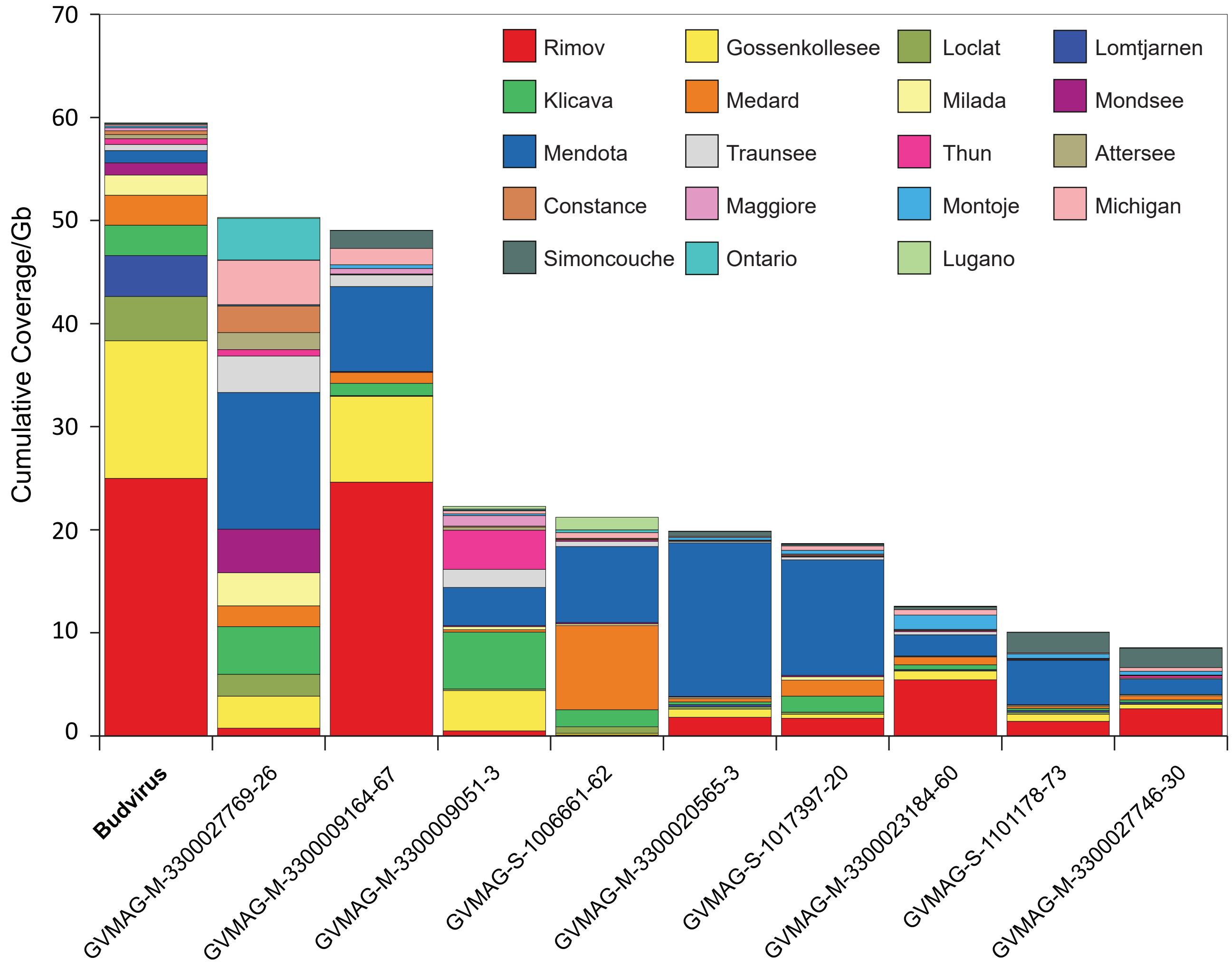

Supplementary Figure S8. Top ten abundant giant viral genomes in the *Budvirus* clade. Cumulative abundances (in coverage/gb) are shown on the y-axis. Legend for lakes is shown at top right.
