# Supplementary material for: Isolation of a widespread giant virus implicated in cryptophyte bloom collapse
Source: ISME J. 2024 Feb 24;18(1):wrae029. doi: 10.1093/ismejo/wrae029 (PMC10960955; doi:10.1093/ismejo/wrae029)
Supplement: Supplementary_Figure_S9 [file supplementary_figure_s9.pdf]

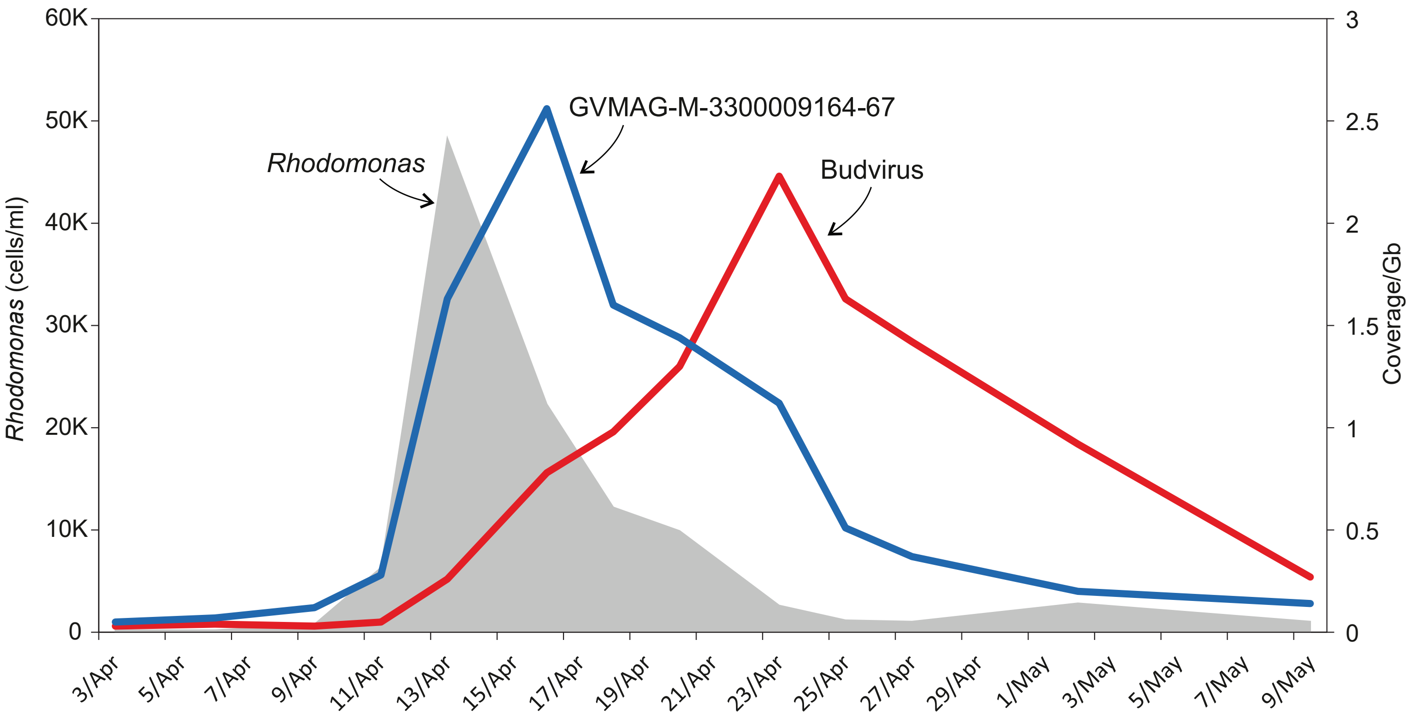

Supplementary Figure S9. Abundance of *Budvirus* and GVMAG-M-3300009164-67 viral genomes in the high-resolution Spring bloom metagenomics dataset from the Rimov reservoir (1st week of April 2018-1st week of May, 2018). Microscopic counts of *Rhodomonas* are shown on the left y-axis and cumulative abundances for the viral genomes (in coverage/gb) are shown on the right y-axis.
