# Supplementary material for: Isolation of a widespread giant virus implicated in cryptophyte bloom collapse
Source: ISME J. 2024 Feb 24;18(1):wrae029. doi: 10.1093/ismejo/wrae029 (PMC10960955; doi:10.1093/ismejo/wrae029)
Supplement: Supplementary_Figure_S10 [file supplementary_figure_s10.pdf]

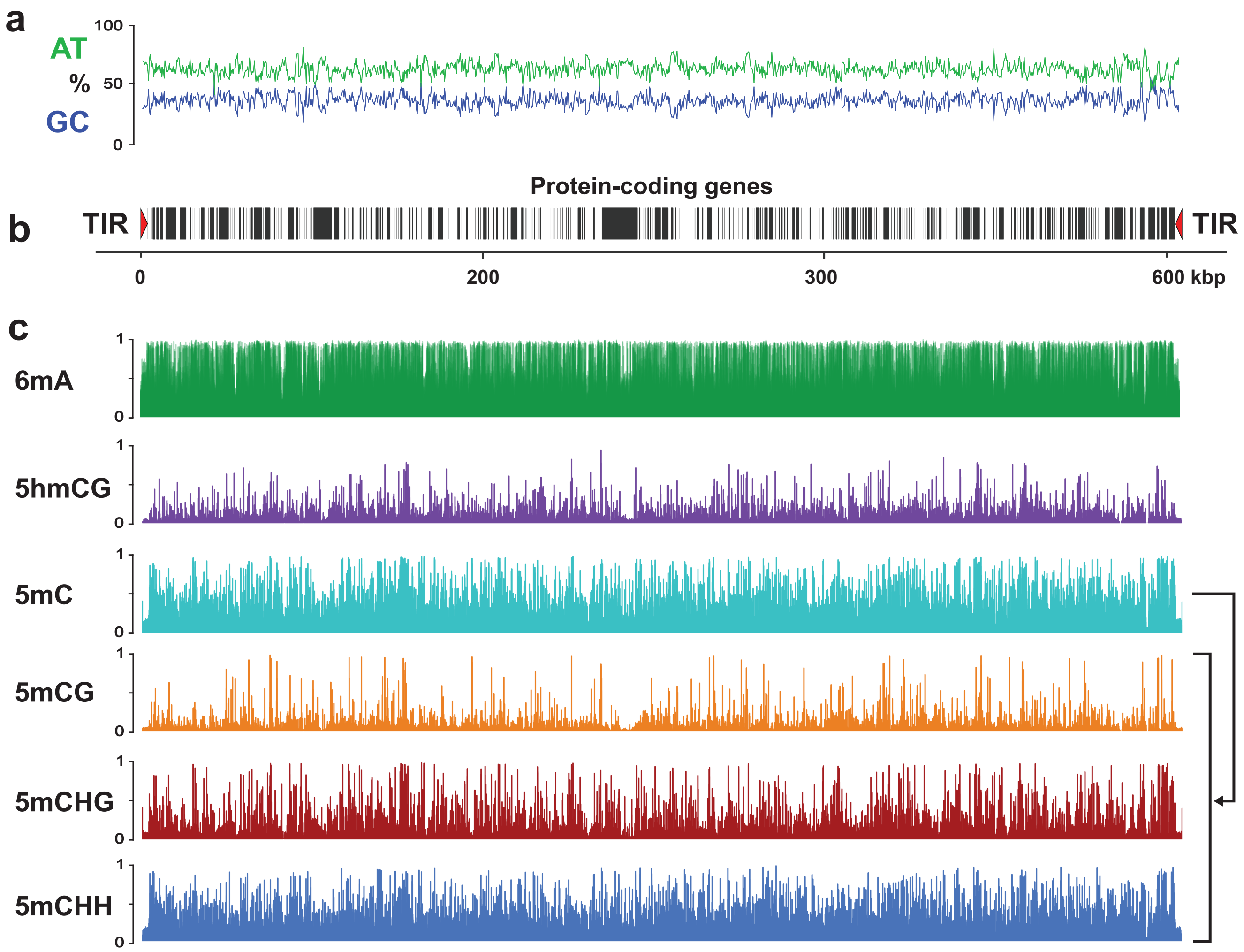

Supplementary Figure S10. Methylation profiles across the *Budvirus* genome.
a. AT (adenine-thymine) and GC (guanine-cytosine) percent composition tracks calculated over 500 bp windows. **b.** Gene density with terminal inverted repeats (TIRs) marked as red triangles. **c.** Methylation tracks for modified A (adenine) and C (cytosine) bases across both strands and height showing fraction of methylated bases at each position. Abbreviations used for methylated/hydroxymethylated base calls: 6mA = 6-methyladenine, 5hmCG = 5-hydroxymethylcytosine (CG-context), 5mC = 5-methylcytosine, 5mCG - 5-methylcytosine (CG-context), 5mCHG - 5-methylcytosine (CHG context; H= A,T or C), 5mCHH - 5-methylcytosine (CHH context).
