# Supplementary material for: Isolation of a widespread giant virus implicated in cryptophyte bloom collapse
Source: ISME J. 2024 Feb 24;18(1):wrae029. doi: 10.1093/ismejo/wrae029 (PMC10960955; doi:10.1093/ismejo/wrae029)
Supplement: Supplementary_Figure_S11 [file supplementary_figure_s11.pdf]

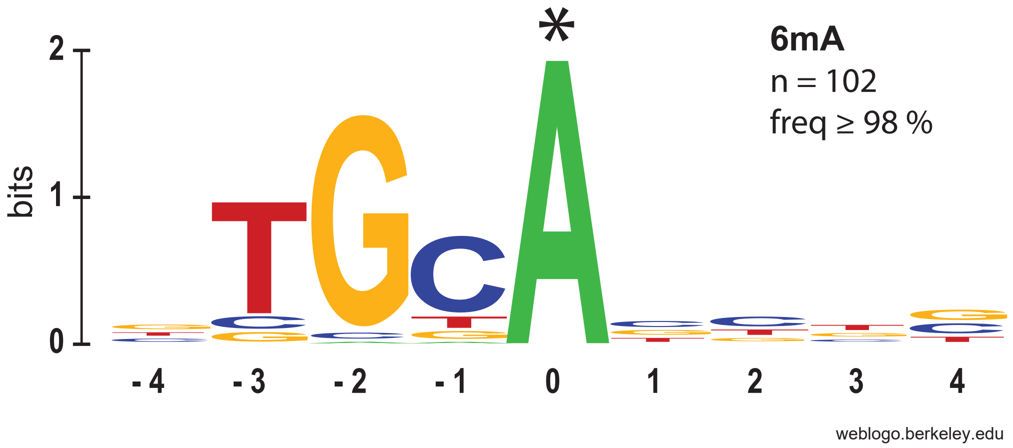

Supplementary Figure S11. DNA logo of sequences flanking methylated adenine residues (6mA) with at least 98% of read basecalls recorded as being methylated. The x-axis indicates distance (in bp) from the site of methylation.
