# Supplementary material for: Isolation of a widespread giant virus implicated in cryptophyte bloom collapse
Source: ISME J. 2024 Feb 24;18(1):wrae029. doi: 10.1093/ismejo/wrae029 (PMC10960955; doi:10.1093/ismejo/wrae029)
Supplement: Supplementary_Information [file supplementary_information.pdf]

Supplementary Information

Isolation of a widespread giant virus implicated in cryptophyte bloom collapse.

Helena Henriques Vieira[1,†], Paul-Adrian Bulzu[1,†,#], Vojtěch Kasalický[1], Markus Haber[1], Petr Znachor[1], Kasia Piwosz[2], Rohit Ghai[1]

[1]Department of Aquatic Microbial Ecology, Institute of Hydrobiology, Biology Centre of the Czech Academy of Sciences, 37005 České Budějovice, Czech Republic.
[2]Department of Fisheries Oceanography and Marine Ecology, National Marine Fisheries Research Institute, 81-332 Gdynia, Poland.
[†]These authors contributed equally to this work

[#]Corresponding author: Paul-Adrian Bulzu, Department of Aquatic Microbial Ecology, Institute of Hydrobiology, Biology Centre of the Czech Academy of Sciences. Na Sádkách 702/7, 37005 České Budějovice, Czech Republic. Email: ✉ bulzupaul@gmail.com

Materials and Methods

Host infection and virus isolation: The strain *Rhodomonas lacustris* NIVA – 8/82 was obtained from the Norwegian Culture Collection of Algae (NORCCA) and cultivated in flasks of 300 ml in WC media [1], at 21ºC, in a dark/light cycle of 16:8 h. A volume of 10 L of surface water (0.5 m) was collected from Římov reservoir (48°50'56" N 14°29'24" E, Czech Republic) and underwent sequential filtration in 5 µm (47 mm, Sterlitech, PES5047100), 1 µm (47 mm, Sterlitech, PCTF1047100), and 0.22 µm filters (47 mm, Sterlitech, PES0247100). Biomass retained on the 0.22 µm filter was diluted in 50 ml of sterilised sample water. The final filtrate passing through the 0.22 µm membrane was concentrated down to 200 ml by tangential flow filtration (100 kDa, 2.5 ft2 cartridge, Millipore, CDUF002LH). Therefore, inocula from two size fractions (1 - 0.22 µm and 0.22 µm to 100 kDa) for virus isolation. *R. lacustris* cultures were inoculated with viral samples from both size fractions. Infection was monitored in 12-well plates, one for each fraction and both plates with three wells for controls. In each well, 3.6 ml of fresh cultures were inoculated with 0.4 ml of viral sample. Positive viral infection was observed by a change in colour from pink to yellow-green, indicating culture collapse, and was confirmed by microscopy (DAPI staining). Nine wells showed viral infection from the larger fraction and only one from the smaller fraction. Infected culture from one of the nine wells was filtered through a 1 µm membrane to remove host cell debris, and the filtrate was purified by reinoculation into fresh *R. lacustris* cultures by serial dilution to extinction (10^{-1} to 10^{-7} dilutions). After four rounds of purification, 100 µl of the isolate was reinoculated into 150 ml of fresh *R. lacustris* culture to produce biomass for DNA isolation. The chosen viral isolate induced the collapse of *R. lacustris* cultures approximately 48 hours after infection, and the viral biomass was promptly harvested following the collapse. To test host specificity, the purified viral isolate was inoculated in four other cryptophyte cultures: *Rhinomonas nottbecki* (K-1855, NORCAA), *Teleaulax amphioxeia* (K-1837, NORCAA), *Storeatula sp.* (K-1488, NORCAA) and C*ryptomonas sp*. (979-4, SAG). In one 48-well plate, eight wells with 1 ml of culture were reserved for each strain: five were inoculated with 100 µl of the viral isolate and three wells were kept as control. The strains were monitored until the natural collapse of the control wells.

***Budvirus* harvesting and DNA isolation:** Infected culture (150 ml) was passed by syringe in 10 ml volumes through a stack of 3 successive filters 1 µm (25 mm, Sterlitech, PCT1025100), 0.45 µm (25 mm, Millipore, 16533K) and finally 0.1 µm (25 mm, Millipore,16553K). Two 0.1 µm filters with equal amounts of biomass were collected and used for DNA isolation with the Quick-DNA HMW MagBead Kit (Zymo, D6060). Before isolation, the filters were cut into 3 mm squares in DNA/RNA Shield reagent (Zymo, R1200) (1 ml), incubated for 1 h RT, treated with Proteinase K (20 µl, 20 mg/ml stock) and SDS 20% 20 µl for 1h at 56˚C. The rest of the DNA isolation protocol for DNA/RNA shield stored samples was followed without modifications. Final elution of DNA was performed with 110 µl of nuclease free water (100 µl recovered). A concentration of 11.4 ng/µl was measured with the Qubit Fluorometer (version 3, Invitrogen) using the Qubit dsDNA HS Assay Kit (Thermo, Q32854). The sample was further concentrated using a vacuum concentrator (Thermo Scientific Savant SpeedVac DNA 130) for 15 minutes at 50˚C resulting in a final volume of 40 µl with a concentration of 28.5 ng/µl. Separate aliquots were prepared for Illumina short-reads sequencing (9 µl sample + 15 µl Elution Buffer EB: 256 ng) and Nanopore MinION long-reads sequencing (31 µl: 883.5 ng).

Transmission electron microscopy: Sample from freshly collapsed culture was collected and allowed to precipitate in 2 ml tubes. A 5 µl aliquot was taken from the bottom of the tube and prepared for transmission electron microscopy. Viruses were observed using the

negative-staining method [2]. Copper grids (T300-Cu; Electron microscopy sciences, USA) were coated with a thin (2-5 nm) carbon layer (formvar 0.3%; SERVA Electrophoresis GmbH, Heidelberg, Germany) and a small volume (10-20 µl) of the infected *Rhodomonas* culture was placed on the top. The samples were briefly (<1 min) stained with uranyl acetate (Lachema n.p. Brno, Czechia), and excess volume was carefully removed from the side by a blotting paper, or the grids were air-dried. Images were captured on a JEOL JEM-1010 microscope (JEOL, Japan) with accelerating voltage 120kV using a CMOS camera XAROSA (EMSIS GmbH, Münster, Germany) 20 MP.

***Rhodomonas* cell counts:** The abundance of *Rhodomonas* was determined from the long-term dataset of the reservoir monitoring programme using standard phytoplankton enumeration methods [3]. Briefly, samples preserved with acid Lugol's solution were enumerated with the Utermöhl method [4]. The preserved sample was thoroughly mixed, and a sub-sample of known volume was placed in a sedimentation chamber. The next day, when the algae had settled to the bottom of the chamber, *Rhodomonas* cells were counted and identified using an inverted microscope (Olympus IMT1).

Illumina and Nanopore sequencing: Illumina short-read paired-end sequencing for DNA was performed on the NovaSeq 6000 platform (Illumina) at Novogene (Hong Kong, China) producing 144,597,504 reads (2 x 150 bp), 21.7 Gbp. Nanopore library preparation, including DNA end repair, and sequencing was done in-house (National Marine Fisheries Research Institute, Gdynia, Poland) according to the SQK-LSK114 workflow on a MinION Mk1C machine. We performed DNA end repair using the NEBNext Companion Module for Oxford Nanopore Technologies (NEB, E7180S) and library preparation using the Ligation Sequencing Kit V14 (SQK-LSK114) with Lambda phage DNA CS (DCS) included for quality control. Sequencing was run on a FLO-MIN114 flow cell for 72h with fast basecalling (Guppy v6.4.6) producing 1,562,507 reads, N50: 8,323 bp, longest read: 145,866 bp, 8.57 Gbp output at a minimum quality threshold of $Q \geq 8$.

Sequence data pre-processing: *Illumina* raw sequencing data was filtered for low-quality bases, reads, and adapters were trimmed using tools provided in the BBMAP software pack v.38.86 (https://sourceforge.net/projects/bbmap/): raw reads were interleaved and quality trimmed by reformat.sh, followed by bbduk.sh (Phred score = 18). Additionally, bbduk.sh was used to remove any PhiX and p-Fosil2 contamination. ***Nanopore*** long-reads were subjected to adapter trimming by Porechop v.0.2.4 [5] followed by length trimming at ≥ 5 kb and $Q \geq 10$ using NanoFilt v.2.8.0 [6].The curated Illumina reads were used to create a Burrows Wheeler Transform (BWT) according to the ropebwt2 (release 187) Construction Approach [7]. Trimmed and filtered Nanopore long-reads were then polished using the Illumina BWT with FMLRC2 v.0.1.8 [8] with a custom, extended, k-mer list (--K 21,59,77,99,129,149). The FMLRC2 polished long-reads (in FASTA format) were filtered again at ≥ 5 kb and saved in a FASTQ file with faux quality scores using reformat.sh. This file was further used as input in the Trycyler v.0.5.4 pipeline [9].

Trycycler assembly reconciliation: We used Trycycler to generate independent assemblies of 26 non-overlapping subsets of the original polished long-reads (16,958 reads/set) (26 is the maximum number of assemblies that Trycycler can handle at a time). To achieve random, non-overlapping read subsets, the option ***subsample*** was used with custom parameters --count 26 --genome_size 1.8m. Each subset of reads was assembled independently using CANU v.2.3 [10] (genomeSize=650,000 useGrid=false -nanopore -corrected). The assemblies were ***clustered*** within Trycycler with default options. Giant virus contigs were identified by scanning all contigs against a custom taxonomy database consisting of GTDB (release 214) combined with eukaryotic and viral sequences from

UniProt (release 2023-01). All identified giant virus contigs were consistently grouped within one cluster which was further processed by trycycler function ***reconcile***. Run in default mode, reconcile assumes that the assembled genomes are circular. Because circularization failed for all viral contigs, a second run was performed with the --linear option. Both runs used the custom options --min_1kbp_identity 0 with the complete polished FASTQ read set. The following steps, ***msa*** - for aligning assemblies, ***partition*** - for recovering reads originating from the target genomes, and ***consensus*** - final sequence generation, were run with default parameters.

Manual curation of *Budvirus* genome: The final sequence generated by Trycycler (609,433 bp) was further extended by manual curation in BioEdit v.7.7 [11] of the MSA produced by Trycycler. The result is a final giant virus consensus genome of 609,674 bp. Almost perfect Terminal Inverted Repeats (TIRs) were identified at the extremities: 4469 bp at the 5' end and 4417 bp at 3p. TIRs were reciprocally compared by building a DNA dot plot using the program Gepard v.2.1[12] (settings: window size = 50, word size = 50).

Basecalling of modified bases: A subset consisting of the first 100 raw FAST5 Nanopore files (4000 reads/file) was chosen for accurate base calling of 6-methyladenine and 5-methylcytosine (6mA and 5mC) as well as 5-hydroxymethylcytosine (5hmC). For optimal performance, the subset of FAST5 files was converted to the POD5 format using Pod5 v.0.2.3. Basecalling was done using Dorado v.0.3.1 (https://github.com/nanoporetech/dorado) in "super-accuracy" (SUP) mode. For 6mA and 5mC basecalling Dorado was used with the Rerio (https://github.com/nanoporetech/rerio) all-context modified base models res_dna_r10.4.1_e8.2_400bps_sup@v4.0.1_6mA@v2 and res_dna_r10.4.1_e8.2_400bps_sup@v4.0.1_5mC@v2 with default parameters (Reads basecalled: 368,309, Reads filtered: 31691). For 5hmCG calling, the same program was run using the dna_r10.4.1_e8.2_400bps_sup@v4.1.0_5mCG_5hmCG@v2 CG-context trained model (Reads basecalled: 399,997, Reads filtered out:3). Basecalled BAM files were filtered based on alignment score (min_q > 20), sorted and indexed using samtools, then converted to bedMethyl format with modbam2bed v.0.9.5 in extensive mode. The bedMethyl files are available in Figshare. In the case of 5mC, modbam2bed was used to generate table files for 3 different sequence contexts (CpG, CHG, CHH). Resulting bedMethyl files were filtered to remove any *nan* entries and those for which the sum of called canonical (Ncano), modified (Nmod) and alternatively modified bases (Naltmod) at the respective genomic position was < 100. Resulting files were used for plotting base modification tracks in R v.4.3.0 packages Gviz v.1.44.0, GenomicRanges v.1.52.0. DNA logos were generated using WebLogo v.2.8.2 (https://weblogo.berkeley.edu/logo.cgi) with sequences covering 6 bp up- and downstream of each modified base passing the threshold frequency for modified base-calling (70 or 90%). The functions slop and getfasta provided within bedtools v.2.29.2 were used to expand and extract sequences flanking methylation sites.

Genome annotation: *Budvirus* encoded proteins were predicted using Prodigal v.2.6.3 in metagenomic mode. The collection of sequences was then locally scanned against the BFD database (https://bfd.mmseqs.com/) using hhblits_omp (settings: -M 50 -n 3 -cpu 80 -cov 20 -norealign). Resulting hhm models were scanned using a local version of hhsearch_omp (-cov 80) against each of the databases: PfamA v.35, NCBI_CD v.3.19, pdb70 (release 220313), scop70 v.1.75, ECOD_F70 (release 20220613), uniprot_sprot_vir70 (release Nov_2021). *Major capsid genes* were identified by scanning predicted proteins with hmmsearch v.3.3 (e-value < 1e-3) against a published set of MCP HMMs covering all major viral groups (Lopez-Garcia et al. 2023; https://figshare.com/articles/dataset/MCP_HMMs/22093253). Identified MCPs (n = 4) as well as the penton minor capsid protein (mCP) were modelled with AlphaFold2 Multimer v.3

using default parameters. Identified capsid genes are presented separately in Supplementary Table S3. Modelled viral capsids are shown in Supplementary Figure S2. Complete Alphafold2 results files are included in Figshare.

Restriction and modification (RM) systems: were investigated by scanning predicted proteins against all annotated restriction endonuclease and methylase entries in the Restriction Enzyme Database (REBASE, downloaded August 2023) using MMseqs (r.14-7e284) easy-search (--search-type 3 --sort-results 1). Results are shown in Supplementary Table S3. Also included is one restriction endonuclease identified only by deep homology searches with online HHpred (https://toolkit.tuebingen.mpg.de/tools/hhpred) against Pfam_A v.35, ECOD_F70 (release 20230309), PDB_mmCIF70_18_Jun, UniProt-SwissProt-viral70_3 (release Nov-2021).

Genome statistics: General genome statistics for *Budvirus* and reference genomes were calculated using the statswrapper.sh subprogram of BBMAP (settings: format=3 gcformat=2). Aragorn v.1.2.38 was used to predict tRNA encoding genes (settings: -t -gc11 -seq -br -fasta -fo). CheckV v.1.0.1 was used to evaluate the genome quality of *Budvirus* and reference genomes with default parameters and the Checkv v.1.5 database.

Variant detection in the environment: All curated paired-end Illumina sequencing datasets (n = 57) generated during a previous sampling campaign of the Římov reservoir [13] were mapped to the assembled *Budvirus* genome using bbwrap.sh with a minimum identity threshold of 95%. All aligned reads passing this threshold from all datasets were merged into one interleaved fastq file (341 Mbp). The merged dataset was mapped back to *Budvirus* using HISAT2 v.2.1.0 [14] with stringent conditions (--sensitive --no-mixed --no-discordant --no-spliced-alignment). A total of 387,849 (23.6%) reads aligned concordantly exactly 1 time and 4248 (0.2%) aligned concordantly >1 times (23.8% overall alignment rate). Samtools v.1.9 [15] was used to convert SAM output to sorted BAM. Variant detection was performed using the FindVariation/SNPs application in Geneious v.9.1.3 (https://www.geneious.com) (settings: min coverage 50, min variant frequency 0.25, p-value calculation method - Exact). Results shown in Supplementary Table S5.

Phylogenomic tree: Gene prediction was performed for *Budvirus* and reference NCLDVs (n = 662) using Prodigal v2.6.3 in metagenomic mode. Predicted proteomes were scanned with hmmsearch v.3.3 (e-value < 1e-5) for a set of 7 proteins (A32, SFII, RNAPL, PolB, TFIIB, TopoII, VLTF3) that were previously benchmarked for their suitability as informative markers for phylogenetic reconstructions of *Nucleocytoviricota* [16,17]. Complementary markers from this set were identified by two different collections of HMM models, one used by viralrecall (https://github.com/faylward/viralrecall) (A32, PolB, VLTF3), the other derived from the GVOG database (GVOGm0013, GVOGm0023, GVOGm0172, GVOGm0461) (https://github.com/faylward/ncldv_markersearch/tree/master/hmm). Recovered markers sequences were aligned using MAFFT v.7.450 in E-INSI mode, alignments were individually trimmed by trimAL v.1.4.rev22 [18] (settings: -gt 0.5 -keepheader) and concatenated by catfasta2phyml.pl (settings: -c -s -v -f). A Maximum-Likelihood phylogenetic tree was constructed based on the concatenated alignment (663 sequences with 5085 columns, 5080 distinct patterns 4987 parsimony-informative, 51 singleton sites, 46 constant sites) using Iqtree2 v.2.2.2.6 [19] (settings: -B 1000 --alrt 1000 -m TEST). The best-fitting evolutionary model chosen by ModelFinder [20] according to the Bayesian information criterion (BIC) was LG+F+I+G4.

Supplementary References

1. Guillard, R. R. L. & Lorenzen, C. J. Yellow-green algae with chlorophyllide c 1, 2. *J. Phycol.* **8**, 10–14 (1972).
2. De Carlo, S. & Harris, J. R. Negative staining and cryo-negative staining of macromolecules and viruses for TEM. *Micron* **42**, 117–131 (2011).
3. Znachor, P. *et al.* Changing environmental conditions underpin long-term patterns of phytoplankton in a freshwater reservoir. *Sci. Total Environ.* **710**, 135626 (2020).
4. Lund, J. W. G., Kipling, C. & Le Cren, E. D. The inverted microscope method of estimating algal numbers and the statistical basis of estimations by counting. *Hydrobiologia* **11**, 143–170 (1958).
5. Wick, R. R., Judd, L. M., Gorrie, C. L. & Holt, K. E. Completing bacterial genome assemblies with multiplex MinION sequencing. *Microb Genom* **3**, e000132 (2017).
6. De Coster, W., D'Hert, S., Schultz, D. T., Cruts, M. & Van Broeckhoven, C. NanoPack: visualizing and processing long-read sequencing data. *Bioinformatics* **34**, 2666–2669 (2018).
7. Li, H. Fast construction of FM-index for long sequence reads. *Bioinformatics* **30**, 3274–3275 (2014).
8. Mak, Q. X. C., Wick, R. R., Holt, J. M. & Wang, J. R. Polishing De Novo Nanopore Assemblies of Bacteria and Eukaryotes With FMLRC2. *Mol. Biol. Evol.* **40**, msad048 (2023).
9. Wick, R. R. *et al.* Trycycler: consensus long-read assemblies for bacterial genomes. *Genome Biol.* **22**, 266 (2021).
10. Koren, S. *et al.* Canu: scalable and accurate long-read assembly via adaptive k-mer weighting and repeat separation. *Genome Res.* **27**, 722–736 (2017).
11. Hall, T. A. & Others. BioEdit: a user-friendly biological sequence alignment editor and analysis program for Windows 95/98/NT. in *Nucleic acids symposium series* vol. 41 95–98 ([London]: Information Retrieval Ltd., c1979-c2000., 1999).

12. Krumsiek, J., Arnold, R. & Rattei, T. Gepard: a rapid and sensitive tool for creating dotplots on genome scale. *Bioinformatics* **23**, 1026–1028 (2007).
13. Kavagutti, V. S. *et al.* High-resolution metagenomic reconstruction of the freshwater spring bloom. *Microbiome* **11**, 15 (2023).
14. Kim, D., Paggi, J. M., Park, C., Bennett, C. & Salzberg, S. L. Graph-based genome alignment and genotyping with HISAT2 and HISAT-genotype. *Nat. Biotechnol.* **37**, 907–915 (2019).
15. Danecek, P. *et al.* Twelve years of SAMtools and BCFtools. *Gigascience* **10**, (2021).
16. Moniruzzaman, M., Martinez-Gutierrez, C. A., Weinheimer, A. R. & Aylward, F. O. Dynamic genome evolution and complex virocell metabolism of globally-distributed giant viruses. *Nat. Commun.* **11**, 1710 (2020).
17. Aylward, F. O., Moniruzzaman, M., Ha, A. D. & Koonin, E. V. A phylogenomic framework for charting the diversity and evolution of giant viruses. *PLoS Biol.* **19**, e3001430 (2021).
18. Capella-Gutiérrez, S., Silla-Martínez, J. M. & Gabaldón, T. trimAl: a tool for automated alignment trimming in large-scale phylogenetic analyses. *Bioinformatics* **25**, 1972–1973 (2009).
19. Minh, B. Q. *et al.* IQ-TREE 2: New Models and Efficient Methods for Phylogenetic Inference in the Genomic Era. *Mol. Biol. Evol.* **37**, 1530–1534 (2020).
20. Kalyaanamoorthy, S., Minh, B. Q., Wong, T. K. F., von Haeseler, A. & Jermiin, L. S. ModelFinder: fast model selection for accurate phylogenetic estimates. *Nat. Methods* **14**, 587–589 (2017).
